# On the Cause of the Circulation of the Blood

**Published:** 1847-02

**Authors:** 


### ECLECTIC DEPARTMENT,

### AND SPIRIT OF THE MEDICAL PERIODICAL PRESS.

---

*On the cause of the circulation of the blood.* By JOHN WM. DRAPER, M. D. *Prof. of Chem. Univ. of New-York.*

The following is, we presume, taken from a late work by Prof. Draper, which we have not yet had the pleasure of seeing, entitled *a treatise on the forces which produce the organization of plants &c.* We find the extract in the Southern Journal of Med. and Pharm. credited to the Lon. Edin. and Dub. Philosoph. Mag. The rationale of capillary circulation appears to us ingenious and plausible.—*Ed. Buff. Jour.*

*Explanation of the General Physical Principle.*—If, in a vessel containing some water, a tube of small diameter be placed, the water immediately rises to a certain point in the tube and remains suspended.

Let the tube be now broken off below that point, and replaced in the cup of water; the liquid rises as before but though it reaches the broken extremity it does not overflow. A capillary tube may raise water to its highest termination, *but a continuous current cannot take place through it.*

Now, suppose a rapid evaporation of the liquid to ensue from the broken extremity of the tube, as fast as the removal of one portion is accomplished others will rise through the tube, and in the course of time the vessel will be emptied. By evaporation from the upper extremity a continuous current is established; a spirit-lamp, with its cap removed, is an example of this fact.

Or, if the liquid which has risen to the upper end of the tube be of a combustible nature, oil for example, and be there set on fire, as the process of combustion goes on a current will be established in the tube, as in a common oil-lamp in the act of burning.

The principle which I wish to draw from these well known facts is, that though ordinary capillary attraction cannot determine a continuous flow of a liquid through a tube, there are very many causes which may tend to produce that result.

If a given liquid occupies a capillary tube, or a porous or parenchymatous structure, and has for that tube or structure at different points affinities which are constantly diminishing, movement will ensue in a direction from the point of greater to the point of less affinity.

Or thus:

If a given liquid occupies a capillary tube, or a porous or parenchymatous structure, and whilst in that tube or structure changes happen to it, which tend continually to diminish its attraction for the surface with which it is in contact, movement will ensue in a direction from the changing to the changed fluid.

*Application of this principle to the Circulation of the Blood.*—Let us now apply these principles to some of the circulations which take place in the human system, and select for that purpose the four leading forms, the systemic, the pulmonary, the portal and the placental circulation.

*The Systemic Circulation.*—The arterial blood, which moves along the various aortic branches, contains oxygen which has been obtained in its passage over the air-cells of the lungs, an oxidation which is indicated by its bright crimson tint. On reaching its final distribution in the tissues, it effects their oxidation, producing heat; and as it loses its oxygen, and receives the metamorphosed products of the tissues, it takes on the blue color characteristic of venous blood.

If now we contrast the relations of arterial and venous blood to the tissues, it is obvious that the former, from the fact that it can oxidize them, must have an intense affinity for them; but the latter, as it is the result of that action after all affinities have been satisfied, must have an attraction which is correspondingly less.

Arterial blood has therefore a high affinity for the tissues; venous blood little or none. But the change from arterial to venous blood takes place in the manner I have just indicated; and therefore, upon the first of the foregoing general rules, motion will take place, and in a direction from the arterial to the venous side.

By the de-oxidizing action of the tissues upon the blood, that liquid ought upon these principles to move from the arteries into the veins, in the systemic circulation. The systemic circulation is therefore due to the de-oxidation of arterial blood.

*The Pulmonary Circulation.*—In this circulation venous blood presents itself on the sides of the air-cells of the lungs, not to carbonaaeous or hydrogenous atoms, but to oxygen gas, which being the more absorbable of the constituents of the air, is taken up and held in solution by the moist walls of those cells. Absorption of that oxygen takes place, and arterialization is the result. The blood from being blue turns crimson.

What now are the relations between venous and arterial blood and oxygen gas? For that gas venous blood has a high affinity, as is shown by its active absorption; but this affinity is satisfied and has ceased in the case of arterial blood.

The change from venous to arterial blood, which takes place on the air-cells which are charged with oxygen gas, ought upon these general principles to be accompanied by movement in a direction from the venous to the arterial side.

The pulmonary circulation is due to the oxidation of venous blood, and ought to be in a direction from the venous to the arterial side.

*The Portal Circulation*—Two systems of forces conspire to drive the portal blood out of the liver into the ascending cava.

1st. The blood which is coming along the capillary portal veins, and that which is receding by the hepatic veins, compared together as to their affinities for the structure of the liver, have obviously this relation—the portal blood is acted upon by the liver, and there are separated from it the constituents of the bile; the affinities which have been at work in producing this result have all been satisfied, and the residual blood over which the liver can exert no action constitutes that which passes over into the hepatic veins. Between the portal blood and the structure of the liver there is an energetic affinity, betrayed by the circumstance that a chemical decomposition takes place and bile is separated; and that change completed, the residue, which is no longer acted upon, forms the venous blood of the hepatic veins.

2nd. The blood of the hepatic artery, after serving for the œconomic purposes of the liver, is thrown into the portal plexus. Hence arises a second force. The pressure of the arterial blood in the hepatic crpillaries upon this is sufficient not only to impel it into the capillaries of the portal veins, but also to give it a pressure in a direction towards the hepatic veins; for any pressure which arises between the arterial blood of the hepatic, and its corresponding venous blood, must give rise to motion towards the hepatic veins, no regurgitation can take place backward through the portal vein upon the blood arriving from the chylopoietic viscera, because along that channel there is a pressure in the opposite direction, arising from the arterial blood of the aortic branches. The pressure therefore arising from the relations of the hepatic arterial blood conspires with that arising from the portal blood, and both together join in giving rise to motion towards the ascending cava.

*The Placental Circulation.*—The umbilical arteries carry in their spiral courses, as they twist round the umbilical vein, the effete blood of the fœtus, and distribute it by their ramifications to the placenta, In that organ it is brought in relation with the arterial blood of the mother, which oxidizes it, becoming by that act deoxidized itself. The fœtal blood now returns along the ramifications of the umbilical vein, and finally is discharged from the placenta by that single trunk.

That this is truly a change similar to that which is accomplished in the adult lungs, is shown by the circumstance that the blood of the umbilical arteries becomes brighter on its passage into the umbilical vein.

As the venous blood of the fœtus is thus oxidized by the arterial blood of the mother, movement must of necesssity ensue in it, on the same principle that it ensues in the adult lung, and must take place in the same direction, that is to say, from the venous to the arterial side.

